# A Multicenter, Open‐Label Study to Assess the Safety of Nebulized Tissue Plasminogen Activator for the Acute Treatment of Pediatric Plastic Bronchitis: The PLATyPuS Trial

**DOI:** 10.1002/phar.70056

**Published:** 2025-09-05

**Authors:** Kathleen A. Stringer, David Goldberg, Sharon Chen, Philip Thrush, Eric M. Graham, Adam Lubert, Jeffrey Myers, Laura McLellan, Thomas Flott, Samya Nasr, Kurt R. Schumacher

**Affiliations:** ^1^ Department of Clinical Pharmacy and the NMR Metabolomics Laboratory, College of Pharmacy University of Michigan Ann Arbor Michigan USA; ^2^ Division of Pulmonary and Critical Care Medicine, Department of Internal Medicine University of Michigan Health System Ann Arbor Michigan USA; ^3^ Division of Pediatric Cardiology, Department of Pediatrics Children's Hospital of Philadelphia Philadelphia Pennsylvania USA; ^4^ Division of Pediatric Cardiology, Department of Pediatrics Lucile Packard Children's Hospital, Stanford University Palo Alto California USA; ^5^ Division of Pediatric Cardiology, Department of Pediatrics Northwestern University Feinberg School of Medicine Chicago Illinois USA; ^6^ Division of Pediatric Cardiology, Department of Pediatrics Medical University of South Carolina Charleston South Carolina USA; ^7^ Division of Pediatric Cardiology, Department of Pediatrics Cincinnati Children's Hospital Medical Center Cincinnati Ohio USA; ^8^ Department of Pathology, Lung and Thoracic Pathology University of Michigan Health System Ann Arbor Michigan USA; ^9^ Division of Pediatric Pulmonology, Department of Pediatrics University of Michigan Health System Ann Arbor Michigan USA; ^10^ Division of Pediatric Cardiology, Department of Pediatrics University of Michigan Health System Ann Arbor Michigan USA

**Keywords:** Brasfield score, congenital heart disease, Fontan physiology, metabolomics, oxygen saturation, spirometry

## Abstract

**Introduction:**

Pediatric plastic bronchitis (PB) is a rare complication of surgically palliated congenital heart disease (CHD). Fibrin casts obstruct airways and can cause respiratory distress. There are no therapeutics approved by the United States Food and Drug Administration to treat PB, but inhaled tissue plasminogen activator (tPA) has been anecdotally used to relieve symptoms. We conducted a phase II open‐label clinical trial to test the safety of inhaled tPA in pediatric PB.

**Methods:**

Patients with an acute exacerbation of PB requiring hospitalization were enrolled to test the safety of an inhaled tPA regimen (5 mg every 6 h). The primary end point was to assess the safety and tolerability of repeated doses of nebulized, inhaled tPA in pediatric patients with acute PB. Safety parameters consisted of clinical laboratories to assess bleeding, which were measured prior to, during, and after tPA treatment. To benchmark efficacy using spirometry and oxygen saturation, children with Fontan‐palliated CHD without a history of PB, with and without protein losing enteropathy (PLE), and healthy children were enrolled in a control arm that did not receive tPA.

**Results:**

Of the 10 patients with PB screened for enrollment, eight qualified for immediate treatment with inhaled tPA. A total of 29 non‐PB participants (PLE, *n* = 8 [10–18 yo]; CHD, *n* = 9 [8–17 yo]; and healthy, *n* = 12 [7–16 yo]) were enrolled. There were no differences in pretreatment clinical blood laboratory values of hemostasis and those during and after treatment with the study drug (primary safety outcome). However, there were four episodes of self‐limiting epistaxis related to the study drug. Inhaled tPA statistically improved oxygen saturation although this was moderate and likely not clinically significant; inhaled tPA did not alter spirometry values.

**Conclusion:**

In this small, phase II study, repeated doses of inhaled tPA in patients with an acute exacerbation of PB did not result in disrupted systemic coagulation or hematological homeostasis or serious bleeding. However, patients should be monitored for localized bleeding. Larger, randomized trials are needed to provide more comprehensive assessments of bleeding risk and to further assess efficacy.

**Trial Registration:**

ClinicalTrials.gov identifier: NCT02315898

## 1 Introduction

Plastic bronchitis (PB) is a very rare, most often pediatric disease characterized by the formation of airway casts which can lead to obstruction of the airways, respiratory distress, and respiratory failure [1, 2]. Although PB has been attributed to a number of different illnesses, in pediatric patients, it is most frequently observed in those who have undergone surgical palliation of congenital heart disease (CHD). Notably, PB is most commonly reported following total cavopulmonary connection (Fontan palliation) in individuals with single‐ventricle CHD [3]. Development of PB in these patients is likely attributable to a number of factors, including abnormal pulmonary lymphatic flow and/or abnormal lymphatic‐airway connections [4]. However, we and others have shown that PB airway casts that form in these children primarily consist of fibrin [4, 5, 6, 7, 8, 9].

Presently, there is no pharmacotherapy approved by the United States Food and Drug Administration for PB, but because PB cast composition is often fibrin, acute exacerbations of the illness have been successfully treated with inhaled fibrinolytic therapy including tissue plasminogen activator (tPA) [5, 6]. To date, this is done anecdotally because there has been no safety or efficacy testing of this treatment. PB‐induced respiratory distress can be severe, often warranting urgent or emergent bronchoscopy for cast removal. As such, there is a significant unmet need for safety testing of inhaled tPA in children with PB.

To address this need, we undertook an open‐label phase II study of inhaled tPA in children with acute exacerbations of PB that necessitated hospital admission. This study was preceded by promising preclinical work that demonstrated repeated doses of tPA could safely be administered to the airways [10, 11]. The primary end point of this clinical trial was to assess the safety and tolerability of repeated doses of nebulized, inhaled tPA in pediatric patients with acute PB. Secondary objectives included the assessment of pulmonary function (spirometry), detection of fibrin degradation products (FDP) in the systemic circulation, and changes in the chest radiograph. Additional secondary outcomes can be found in the Supporting Information. As a tertiary end point, we conducted biomarker discovery using blood metabolomics to provide clues about inhaled tPA drug response and to identify metabolites that differentiate patients with PB from healthy children and those with another complication of CHD surgical palliation, protein losing enteropathy (PLE).

## 2 Methods

### 2.1 Human Subjects

The Investigational New Drug (IND) protocol (IND#119678) and associated informed consent and assent forms were approved at each clinical site (Children's Hospital of Philadelphia (CHOP), institutional review board (IRB) 17‐014421; Cincinnati Children's Hospital, IRB ID 2017‐5603; Ann & Robert H. Lurie Children's Hospital of Chicago, IRB 2018‐1914; Medical University of South Carolina, Pro00072570; Stanford University, protocol #45157; and the University of Michigan, HUM00111748) in advance of patient enrollment. The University of Michigan served as the data coordinating center (HUM00141246) and the study was preregistered on ClinicalTrials.gov prior to patient enrollment (NCT02315898).

The study was conducted following the principles of the Declaration of Helsinki and in accordance with US Federal Policy for the Protection of Human Subjects. All site principal investigators and study personnel completed required protection of human subjects education and training. All participants and their legal guardian (as applicable) signed informed consent prior to the initiation of any study‐related procedures; participants between the ages of 10–14 years of age signed assent; children > 14 years of age could co‐sign the informed consent document with their legal guardian.

### 2.2 Study Protocol

This was a phase II open‐label study that enrolled patients with an acute exacerbation of PB eligible for treatment with inhaled tPA (alteplase, Activase, Genentech, South San Francisco, CA USA). Inclusion and exclusion criteria for the treatment arm are provided in Table 1. In a separate arm, to benchmark efficacy using spirometry and oxygen saturation, and safety using clinical laboratories, age‐range matched healthy subjects and patients with Fontan circulation with uncomplicated Fontan physiology and no history of PB, other Fontan‐associated complications (e.g., hepatopathy, PLE), or other concomitant illnesses (e.g., asthma) were enrolled. As an additional exploratory arm, we enrolled patients with Fontan physiology and no history of PB who had a diagnosis of PLE defined as clinically symptomatic hypoproteinemia attributed to enteral protein loss. These single‐visit, outpatient participants were not treated with tPA. The single research visits for these participants consisted of the collection of blood and urine specimens, assessment of clinical laboratories, spirometry, and a physical exam. Additional information about inclusion–exclusion criteria can be found in the Supporting Information. Inclusion–exclusion criteria were modified over the course of the study, most notably in response to the SARS‐CoV‐2 pandemic.

**TABLE 1:** Inclusion–exclusion criteria for PLATyPuS trial treatment arm.

*Inclusion criteria* Patients who present with an acute exacerbation of PB,^a^ defined as the expectoration of, or a bronchoscopy retrieved, fibrin PB cast that causes acute respiratory distress (e.g., severe coughing, difficulty breathing, dyspnea) and are: ≥ 5 years of age but ≤ 24 years of age and weigh at least 18.6 kg (41 lbs) have CHD and a history of PB with previous airway cast production^a^ able to use a mouthpiece nebulizer
*Exclusion criteria* Known contraindication(s) to the use of tPA Body weight > 100th percentile or BMI > 30 kg/m^2^ Known cystic fibrosis Currently receiving dornase‐alfa and/or inhaled unfractionated or low molecular weight heparin and/or a direct acting oral anticoagulant (e.g., dabigatran, rivaroxaban) Protein losing enteropathy Liver dysfunction (defined as ≥ 3× the normal levels of one or both liver transaminases, AST and ALT) Need for concomitant intravenous or subcutaneous anticoagulation with resulting anti‐Xa levels > 0.5 (low molecular weight heparins) or > 0.3 (unfractionated heparin) International normalized ratio (INR) > 2.0 if not receiving warfarin Patients being actively treated for thrombosis Concomitant use of a thienopyridine class antiplatelet agent (e.g., clopidogrel) A platelet count of < 100,000 platelets/μL A hematocrit < 30% Gross hematuria on screening urinalysis Pregnant or lactating women (negative pregnancy test required for girls/women of child‐bearing potential at the time of inhaled tPA administration). All women of child‐bearing potential must be willing to practice appropriate contraception throughout the study. *Added subsequent to the SARS CoV‐2 pandemic*: 15 Patients who are known positive for or are hospitalized with COVID‐19 caused by the new coronavirus, SARS CoV‐2, at the start of the treatment phase. 16 Suspected or active concurrent infectious illness.

Abbreviations: ALT, alanine aminotransferase; AST, aspartate aminotransferase; BMI, body mass index; CHD, congenital heart disease; COVID‐19, novel coronavirus disease‐2019; PB, plastic bronchitis; SARS CoV‐2, severe acute respiratory syndrome coronavirus 2; tPA, tissue plasminogen activator.

^a^ For patients without CHD that presented with an acute exacerbation of PB, defined as the expectoration of, or a bronchoscopy retrieved, fibrin PB cast that causes acute respiratory distress (e.g., severe coughing, difficulty breathing, dyspnea) or a history of PB with pathologic evidence of fibrin airway cast production, either a cast sample (at least 2 cm [~0.8 in.]) or a pathology report that documented PB cast fibrin content had to be submitted to the University of Michigan pathology core.

### 2.3 Study Drug

Study drug (5 mg) was administered by nebulizer every 6 h for up to 72 h (12 doses). This dose was chosen based on site investigators' extensive experience with using inhaled tPA to treat children with PB and our preclinical data that supported a 6‐h dosing interval [12]. Each dose was prepared at each clinical site by the institution's respective investigational drug service pharmacy. It was supplied by Genentech to a drug depot service (Fisher Clinical Services, Mt. Prospect, IL USA) that subsequently shipped it to each clinical site. Additional details about study drug administration can be found in the Supporting Information.

Participants were followed up to 24 h after the completion of the administration of the last dose of inhaled tPA, whether study drug or that administered as part of clinical care, or up to hospital discharge, whichever occurred first. They were asked to return for a visit 30 days (±7 days) after the completion or termination of inhaled tPA for final safety laboratories.

### 2.4 Safety Assessment

Baseline screening procedures for patients with PB and healthy, PLE and CHD control participants included a physical exam (with a neurological exam for patients with PB), spirometry, pulse oximetry, and blood samples for clinical laboratory tests (hematology, coagulation, and urinalysis), and biomarker discovery (metabolomics), a chest radiograph (PB only; PA posterior–anterior and lateral, as feasible); a negative urine pregnancy test was required for girls with PB of child‐bearing age. Safety assessment parameters consisted of hematocrit, platelet count, fibrinogen, FDP, activated partial thromboplastin time (aPTT), international normalized ratio (INR) and urinalysis to assess hematuria. These parameters were reassessed daily during tPA treatment, at hospital discharge, and at the close‐out visit (day 30). The primary safety end points were the development of new, active systemic and/or pulmonary hemorrhage and/or new hematuria (defined as gross hematuria). The definitions of pulmonary and nonpulmonary hemorrhage are shown in Table 2 [13]. Study data were entered by each site and managed using REDCap electronic data capture tools hosted at the University of Michigan [14, 15].

**TABLE 2:** Definitions of pulmonary and nonpulmonary hemorrhage.

Grade	Defined by
Grading system for pulmonary hemorrhage^a^	
1. Mild (scant)	Hemoptysis of < 5 mL of bright red blood for which intervention is not indicated
2. Moderate	Up to three episodes in a 24‐h period of hemoptysis of > 5 mL ≤ 100 mL of bright red blood or a single episode of > 100 to < 240 mL for which medical intervention is necessary and a radiologic assessment (e.g., chest x‐ray) should be considered
3. Severe	Hemoptysis of ≥ 240 mL and/or suspected pulmonary bleeding for which transfusion, radiologic, endoscopic, or operative intervention is indicated (e.g., hemostasis of bleeding site), or there is life‐threatening respiratory or hemodynamic compromise for which intubation or urgent intervention is indicated
Grading system for other (nonpulmonary) hemorrhage	
1. Mild	Nonactionable bleeding that does not require treatment by a health care professional
2. Moderate	Any overt, actionable sign of bleeding (e.g., gross hematuria) that is more than clinically expected but does not meet the severe criteria below; meets at least one of the following: (1) requires nonsurgical, medical intervention by a health care professional; (2) prolongs hospitalization or an increased level of care; (3) prompts evaluation (e.g., bronchoscopy, imaging)
3. Severe	Overt bleeding with a bleeding related drop of 20% or more in pretreatment hematocrit that cannot be attributed to volume overload or an overt bleeding event that requires transfusion, surgical intervention or the use of intravenous vasoactive agents. Intracranial hemorrhage or intraocular bleeding that compromises vision.

^a^ Adapted from Common Terminology Criteria for Adverse Events v.4.0 (https://ctep.cancer.gov/protocolDevelopment/electronic_applications/ctc.htm) and reference [11].

### 2.5 Clinical Laboratories

Clinical laboratory values were reported from the clinical pathology laboratory at each clinical site. All are Clinical Laboratory Improvement Amendments‐certified and College of American Pathologists‐accredited laboratories. Except for FDP values, normal value ranges were consistent across sites and were used by each site's principal investigator (PI) to assess clinical safety.

### 2.6 Adverse Events

Adverse events (AE) were categorized as expected (yes/no; those listed in the package insert or investigator brochure), related to study drug (yes/no), and graded based on severity. If the AE was related to study drug and while the participant was still receiving study drug, the treating physician had the option to reduce the dose by temporarily withholding treatment or withdrawing treatment altogether.

### 2.7 Measurement of Pulmonary Function and Oxygen Saturation

Spirometry was done in accordance with the American Thoracic Society guidelines [16] in patients with PB prior to, during, and after inhaled tPA treatment. Spirometry measurements were acquired from control participants during their single study visit. Oxygen saturation was assessed by pulse oximetry prior to and after each inhaled tPA dose and at the time of spirometry for both PB and control participants.

### 2.8 Chest Radiograph Scoring

Chest radiographs were obtained in patients with PB prior to tPA treatment (Day 1) and at the end of the study. Posteroanterior and lateral images were scored by a pulmonologist (SN) using the Brasfield scoring system [17]. This scoring system includes assessment of five categories: air trapping (0 = absent to 4 = most severe), linear markings (0 = absent to 4 = most severe), nodular cystic lesions/bronchiectasis (0 = absent to 4 = most severe), lobar involvement (0 = absent to 5 = large lesions), and overall severity, where a score of 0 represents normal, and a score of 5 represents the most severe pulmonary involvement. Scores for each category are subtracted from 25, which represents a normal chest radiograph such that lower Brasfield scores represent more remarkable findings.

### 2.9 Metabolomics

As a tertiary outcome, whole blood samples were assayed by ^1^H‐nuclear magnetic resonance (NMR) spectroscopy to generate metabolomics data. Blood samples were collected from patients with PB prior to, during tPA treatment, and at hospital discharge. Details of the metabolomics methods and the results of additional assays are described in the Supporting Information.

### 2.10 Statistics

The primary analysis consisted of the comparison of pretreatment safety laboratory values to each subsequent time point in PB, tPA‐treated patients using a mixed‐effects analysis followed by a Holm‐Šídák's multiple comparisons test. Other parameters were evaluated by parametric tests as appropriate. Differentiating blood metabolites were identified by comparing the mean transformed value of each metabolite in patients with PB, Fontan, and PLE controls to those of healthy controls using ANOVA followed by a post hoc Holm‐Šídák's multiple comparisons test. Statistical tests were performed, and figures, unless otherwise stated, were generated in PRISM (Version 10.4.0, GraphPad Software, Boston, MA USA).

## 3 Results

### 3.1 Study Population

**FIGURE 1:**
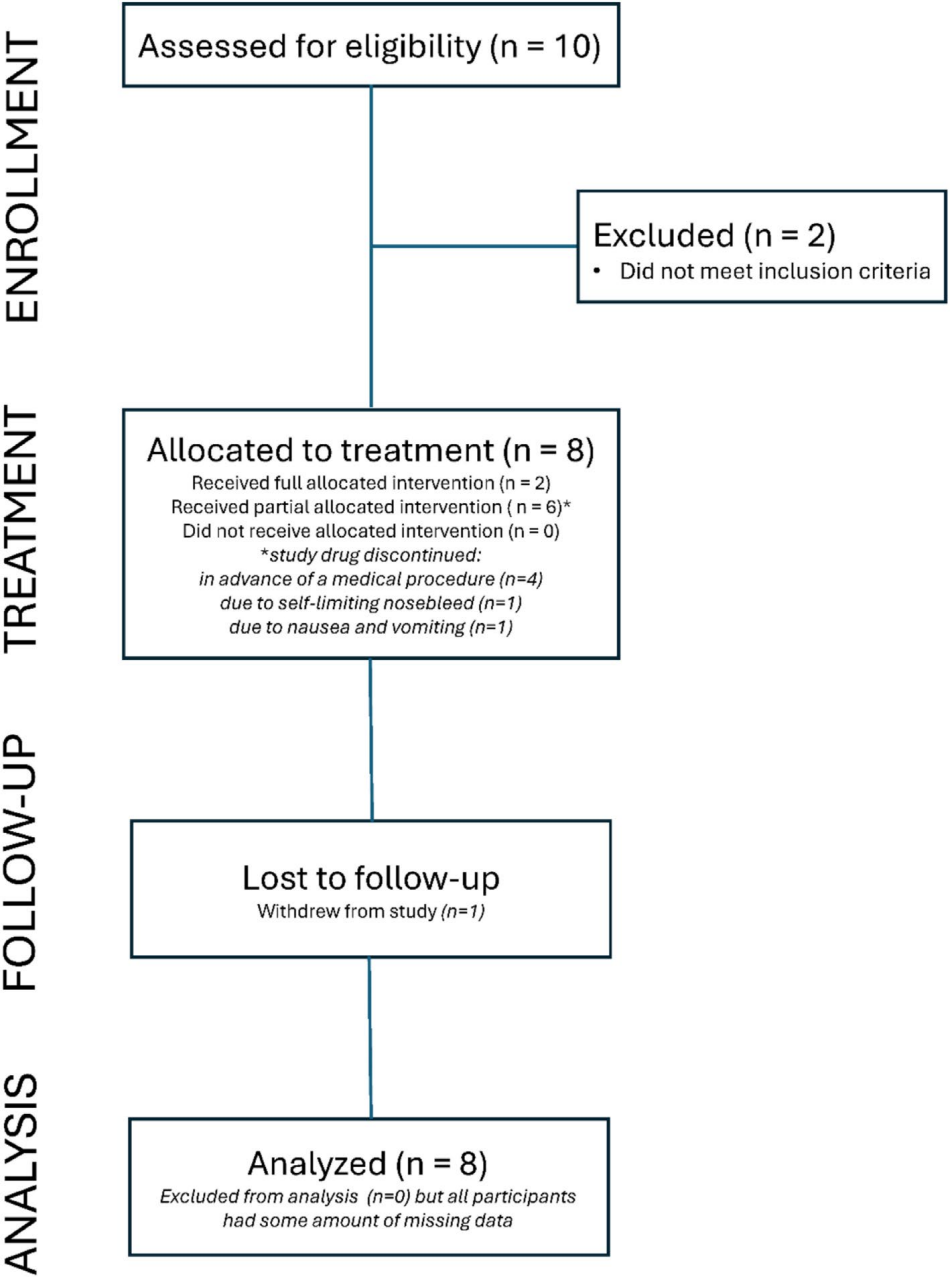
Consort diagram for tissue plasminogen activator treatment arm.

A total of 10 patients (6–18 yo) with a confirmed history of PB were screened for enrollment into the tPA treatment arm of the study (Figure 1) between 2018 and 2023. The study paused from March to September 2020 during the SARS CoV‐2 pandemic. Of these, eight patients qualified for immediate treatment with inhaled tPA; two patients were not eligible for treatment because they did not manifest an acute exacerbation of PB. A total of 29 non‐PB participants (PLE, *n* = 8 [10–18 yo]; uncomplicated Fontan, *n* = 9 [8–17 yo]; and healthy, *n* = 12 [7–16 yo]) were enrolled. The demographics and clinical characteristics of study participants are shown in Table 3.

**TABLE 3:** Participant demographics.

Overall number of participants	Fontan (PLE (−)) + plastic bronchitis (nebulized tPA treated)	Fontan (PLE (+)) controls	Fontan (PLE (−)) controls	Healthy controls	Total
	8	8	9	12	37
Mean age (SD); years	12 (4)	13 (3)	14 (3)	12 (2)	13 (3)
Sex (% female)	50	13	38	33	32
Mean BMI percentile (SD)^a^	86 (12.7)	60.4 (32)	40.7 (14.8)	62.7 (31.4)	63.1 (28.6)
Mean BSA (SD); m^2^	1.4 (0.3)	1.2 (0.5)	1.4 (0.3)	1.4 (0.3)	1.4 (0.3)
Underlying primary congenital heart abnormality					
Single Ventricle (*n*)	8	6	9	n/a	23
HLHS (*n*)	5	1	4		10
Tricuspid atresia (*n*)	3	1	1		5
Unbalanced atrioventricular canal defect (*n*)		3	2		5
D‐loop double outlet right ventricle with two ventricles (*n*)		1			1
Heterotaxia syndrome (*n*)			2		2
Double outlet right ventricle		1			1
Unknown		1			1
Surgical physiology					
Fontan (*n*)	7	8	9	n/a	24
Glenn (*n*)	1			n/a	1
History of PB prior to study enrollment (*n*)	8	0	0	0	8
Mean age at PB diagnosis (SD); years	9.3 (3.8)	n/a	n/a	n/a	
Ethnicity/Race					
No. Hispanic or Latino (%)	2 (25)	2 (25)	0 (0)	2 (17)	6 (16)
No. White (%)	5 (63)	4 (50)	8 (89)	10 (83)	27 (73)
No. other or unknown	1 (12)	2 (25)	1 (11)^b^	0 (0)	4 (11)

Abbreviations: BMI, body mass index; BSA, body surface area; HLHS, hypoplastic left heart syndrome; n/a, not applicable; PLE, protein losing enteropathy; SD, standard deviation.

^a^ BMI percentile was calculated using the CDC's Peditools (https://peditools.org/growthpedi/).

^b^ This patient identified as Asian.

### 3.2 Primary Outcome

#### 3.2.1 Safety Measurements

Inhaled tPA was well tolerated by all PB patients. The mean SD number of administered 5‐mg doses was 9 (3.7) and the cumulative dose (mg) of tPA was 45 (18.5). Two patients had three doses that were only partially completed. These were not included in the total dose calculation since it was not possible to determine the amount of study drug administered. The need to discontinue inhaled tPA in advance of a procedure (percutaneous lymphatic embolization) was the most common reason for not completing the 12‐dose regimen.

**FIGURE 2:**
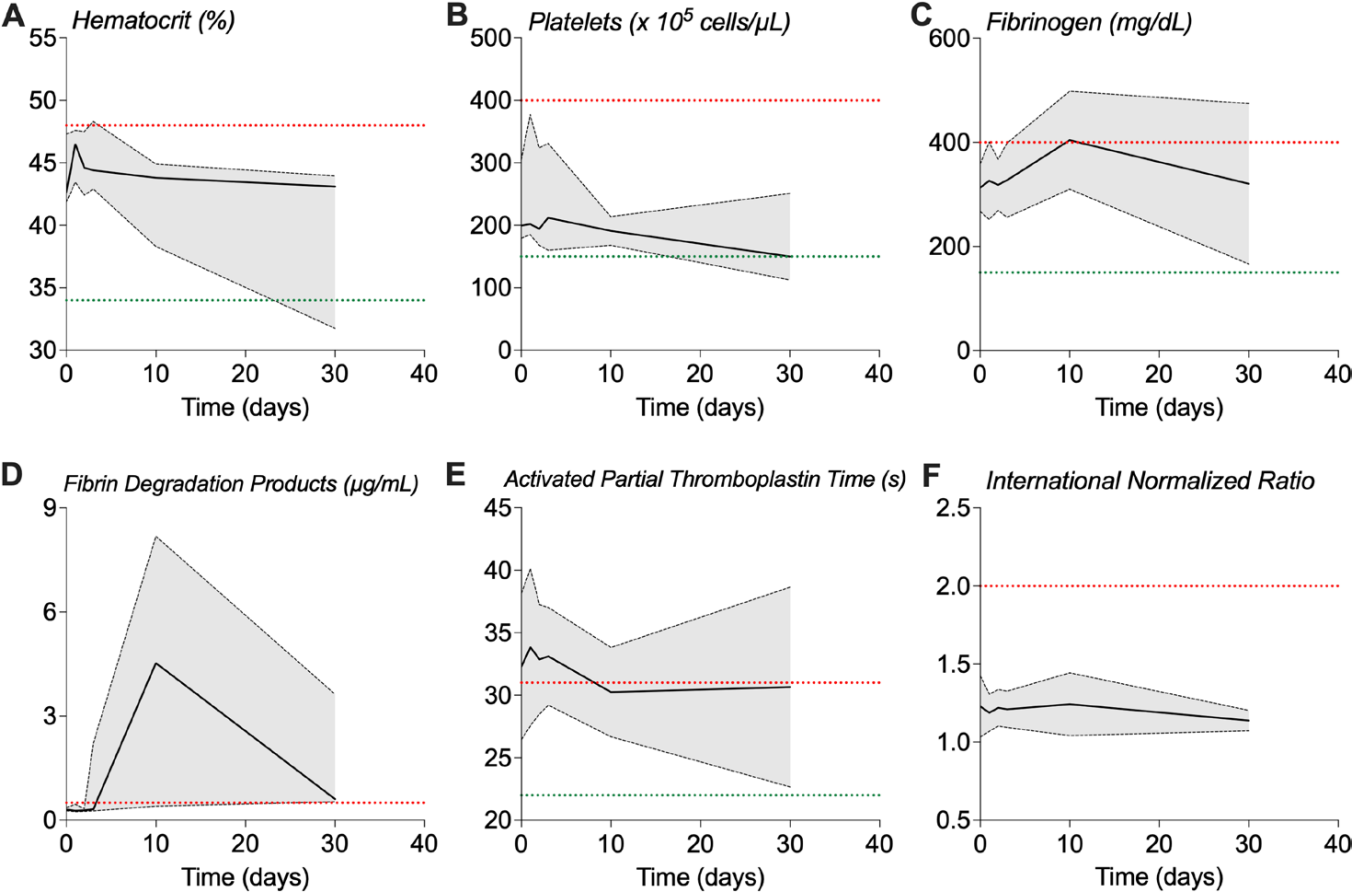
Safety clinical laboratory values prior to, during, and following inhaled tPA treatment in patients with acute plastic bronchitis. Clinical hematologic, (A) hematocrit, (B) platelets, and coagulation, (C) fibrinogen, (D) fibrin degradation products, (E) activated partial thromboplastin time, (F) international normalized ratio, were evaluated prior to, during, and following 72 h of inhaled tPA treatment. Quantitative values of fibrin degradation products (or D‐dimer) (D) are not plotted for two patients because these values were not quantitatively reported (see Table S1). All laboratory values were evaluated by a physician and categorized as within normal limits, clinically significant, or clinically insignificant. Although some values were categorized as clinically significant, they were deemed unrelated to study drug administration. The data are presented as the median value (IQR) and the lower and upper reference values for each parameter (as applicable) is designated by green and red dotted lines, respectively, as applicable. IQR, interquartile range.

There were no differences in pretreatment clinical safety blood laboratory values and those during and after treatment with study drug (Figure 2 and Table S1). Results of urinalyses were unchanged for all patients with PB. Collectively, these findings provide evidence that inhaled tPA did not result in systemic alterations in hemostasis. There were no study drug‐related serious adverse events (SAE), three patients experienced AEs categorized as nonpulmonary hemorrhage (grade 1; see Table 2). These were four self‐limiting episodes of epistaxis in three patients related to study drug. Of these, three events were mild and did not result in discontinuation of study drug. The fourth event (and second for one patient) occurred several hours after a dose of study drug and was clinically relevant, although still self‐limited. Given the possible risk of additional bleeding, study drug was discontinued, with no subsequent sequelae from this event. Information about other AEs can be found in Table S3.

### 3.3 Secondary Outcomes

#### 3.3.1 Oxygenation

Paired oxygen saturation (SpO_2_) values as determined by pulse oximetry were available from seven of the eight patients with PB. Inhaled tPA treatment mildly improved oxygenation in patients with PB (mean (SD): predose, 91.2% (4.1) vs. postdose, 91.9% (4.5); *p* = 0.04 by paired Student's *t*‐test). As expected, baseline SpO_2_ values in patients with Fontan physiology were lower compared to healthy controls (Figure S1).

#### 3.3.2 Spirometry

**FIGURE 3:**
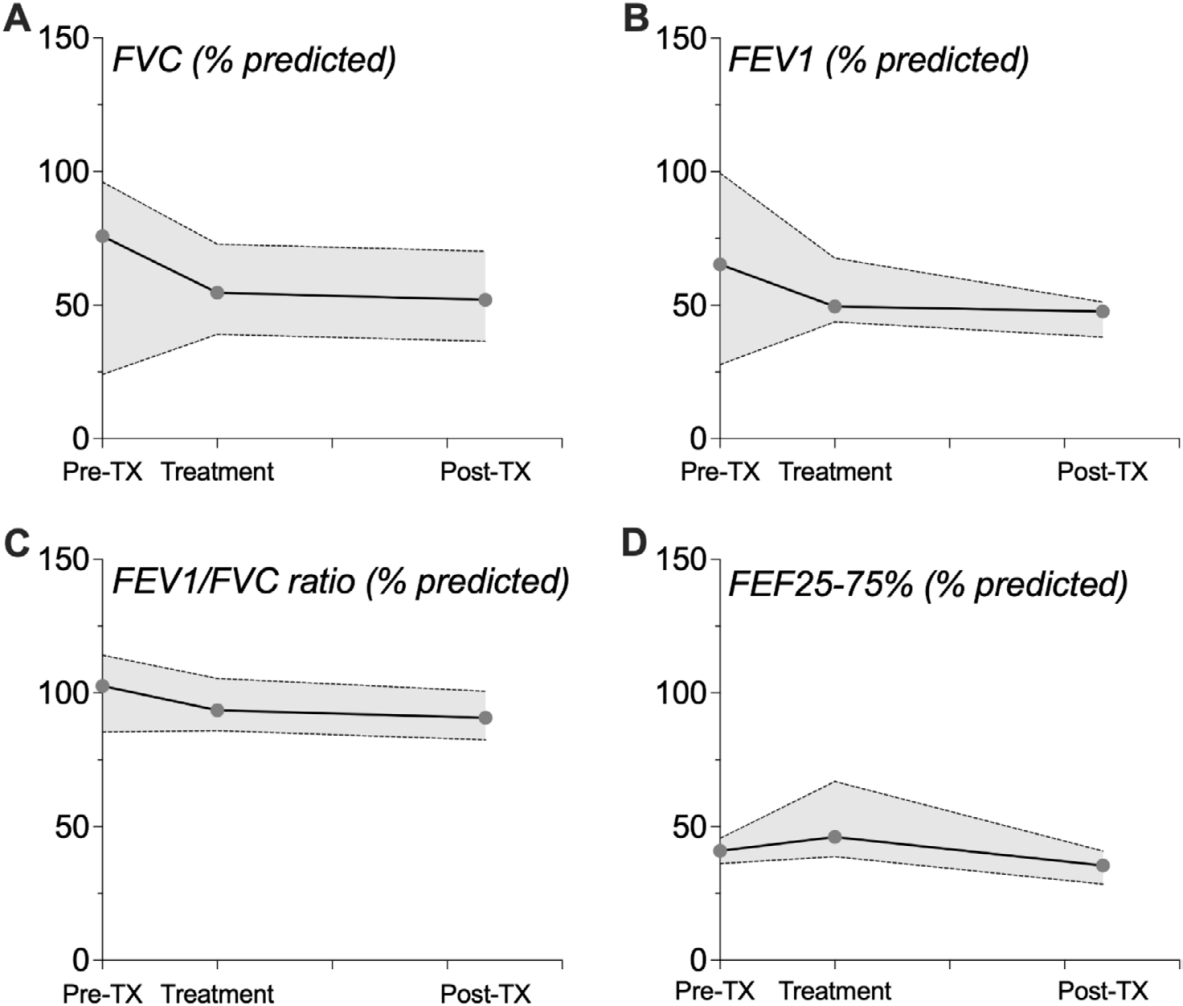
The impaired pulmonary function in children with plastic bronchitis was not improved by treatment with inhaled tPA. Combined (averaged) pretreatment, inhaled tPA treatment (Days 1–3), and posttreatment (hospital discharge and 30‐day follow‐up visit) spirometry values. Since only two to three values for each parameter were available for the pretreatment time point, to assess inhaled tPA effect on spirometry, the statistical (Mann Whitney test) comparison was made between treatment and posttreatment measurements. Data are plotted as the median (IQR) of pretreatment (*n* = 2–3), treatment (*n* = 13, representing seven plastic bronchitis patients), and posttreatment (*n* = 6, representing 6 plastic bronchitis patients) values. Reference values for each parameter were generated from the healthy control (*n* = 12) cohort. FEF25%–75%, forced mid‐expiratory flow; FEV_1_, forced expiratory volume in 1 s; FVC, forced vital capacity; IQR, interquartile range. Also see Table S2.

Due to an insufficient number of baseline (pretreatment) measurements (*n* = 2–3) and challenges to consistently obtain daily spirometry measurements during inhaled tPA treatment, we pooled and averaged measurements made during treatment (Days 1–3) to determine whether tPA treatment influenced pulmonary function and statistically compared them to values acquired posttreatment (hospital discharge and Day 30). Inhaled tPA did not change the percent predicted values for forced expiratory volume in 1 s (FEV_1_), forced vital capacity (FVC), FEV_1_/FVC ratio, or forced expiratory flow at 25%–75% (FEF25%–75%) (Figure 3). Compared with healthy control participants, lung function was impaired in patients with PB. Overall, the pulmonary function testing showed a restrictive pattern which could be due to PB‐induced restriction in the airways (Figure S2). There was evidence of small airway obstruction in all participants with Fontan physiology; this was more pronounced in children with PB.

#### 3.3.3 Chest Radiographs

Images obtained from patients with acute PB were scored using the Brasfield criteria by a co‐investigator experienced with the technique (SN). All patients with PB had remarkable findings (Figure S3) that were not changed by tPA treatment.

Information about additional secondary outcome measures can be found in the Supporting Information.

### 3.4 Tertiary Outcome

#### 3.4.1 Metabolomics

Whole blood metabolomics data were generated by ^1^H NMR spectroscopy at the University of Michigan's Biochemical NMR Core as previously described [18] and these results are detailed in the Supporting Information.

## 4 Discussion

In this small but first study in pediatric patients with acute exacerbations of PB, we found that repeated doses of the commercially available formulation of tPA can be delivered to the airways by nebulization without disrupting systemic coagulation. There was no evidence that the regimen of 5 mg of inhaled tPA every 6 h led to disruption in systemic hematological or coagulation homeostasis, and there were no serious bleeding events. However, there were several episodes of self‐limited, localized bleeding (i.e., epistaxis). Given being underpowered for the study's primary end point, larger trials are needed to provide a more comprehensive assessment of localized bleeding risk. In aggregate, these findings are consistent with reported anecdotal clinical experience with inhaled fibrinolytics for PB treatment that demonstrated feasibility [5, 6, 19, 20, 21, 22, 23, 24] and preclinical work in which repeat administration of nebulized tPA to the airways was well tolerated [11]. They also provide clear new evidence and corroborate prior findings [25] that inhaled tPA is not absorbed systemically, which should minimize the concern for systemic bleeding. This information is important to clinicians in guiding therapy for patients who may be critically ill.

In addition to safety, we aimed to make assessments of inhaled tPA efficacy by measuring the frequency of cast expectoration, size, and composition as surrogates of airway cast burden. We also assessed oxygenation and spirometry during and after treatment. Despite presenting with airway cast production, none of the patients with PB continued with in‐hospital expectorated cast production following the initiation of inhaled tPA. This could be attributed to the efficacy of the regimen, although without a negative (placebo) control comparison, we cannot be confident that this is the case. Furthermore, the overall oxygenation, as measured by pulse‐oximetry, was not reduced by acute exacerbations of PB, so clinically relevant improvements in oxygenation with tPA treatment could not be detected. Notably, patients with PB had pulmonary dysfunction and chest radiographic findings that were not improved by inhaled tPA treatment. This may be due to radiographic findings resulting from chronic changes in the lungs, as opposed to acute fibrin casts, which would not be expected to change with tPA. Collectively, the use of these parameters for the assessment of efficacy was hampered by the study's small sample size and the absence of a control arm. A larger, randomized trial will be needed to robustly test the utility of these end points and evaluate the efficacy of inhaled tPA in PB.

We focused our study on pediatric PB, which most commonly occurs in association with CHD that has been surgically palliated by the Fontan procedure. In this population, the airway cast composition is consistently fibrinous [4, 7, 8] and plasminogen has been shown to be present [9]. This cast composition makes this type of PB amendable to treatment with a plasminogen activator like tPA and explains why inhaled fibrinolytics have been used for symptom relief. We acknowledge that there are other types of PB, including those that can occur in adults with airway disease (e.g., asthma) or recent viral infections, which result in airway casts that are not uniformly composed of fibrin [26]. This illustrates the importance of pathologic confirmation of cast composition before the initiation of inhaled fibrinolytic therapy [7].

This study also permitted the comparison of patients with PB to healthy controls and other groups of children with Fontan physiology without PB but with another complication, PLE [1, 27]. As expected, compared with healthy controls, these children had lower levels of oxygen saturation but also had evidence of small airway obstruction (reduced FEF25%–75%) which was the most pronounced in children with PB. To the best of our knowledge, this finding has not been previously reported. In an exploratory aim, we compared the whole blood metabolomics signatures of children with PB or PLE benchmarked to healthy participants. Patients with PLE had the most disruption in metabolism, while patients with PB had a unique profile that included lower concentrations of the antioxidant, glutathione. The significance of these findings is uncertain but, in aggregate, the metabolomics signature of patients with complications of Fontan physiology may help inform directions for future work.

We acknowledge that there are limitations of our study, the most prominent of which is the study's small sample size; we did not meet our enrollment goal of 11 patients with PB. To address this problem, we avoided multiple comparisons across groups and used healthy controls as the reference group. Plastic bronchitis is an extraordinarily rare disease [3]. Despite utilization of six clinical sites, we did not meet our enrollment goal, and our recruitment efforts were further compromised by the SARS‐CoV‐2 pandemic, which paused the study for 7 months. Furthermore, since the inception of this clinical trial, much has been learned about the etiology of PB, and advancements have been made in its treatment. Specifically, lymphatic flow disorder, characterized by an abnormal or dysregulated circulation of lymph fluid, has been recognized as the primary cause of PB in children with surgically palliated CHD [28]. Communication between the thoracic lymphatic system and the airways leads to the formation of airway casts. These revelations have led to advancement of the use of percutaneous lymphatic embolization as a highly effective intervention for PB treatment [29]. Nevertheless, this procedure is only performed at specialized centers, making therapeutic interventions like inhaled tPA a viable option as well as a bridging strategy for patients who may need to wait for embolization.

In conclusion, this study provides evidence that a 5‐mg dose of nebulized tPA repeatedly administered every 6 h for up to 12 doses is well tolerated and does not alter systemic coagulation or hematological homeostasis. Patients should be closely monitored for localized bleeding (e.g., epistaxis) as further evidenced by a prior study in critically ill adult patients who received higher doses and more prolonged dosing of inhaled tPA [25]. Additional findings, such as the extent of small airway obstruction and metabolomics signatures in children with PB and PLE, introduce new avenues of investigation and pave the way for additional work to optimize therapeutic options for and to further understand the physiologic complexity of children with Fontan physiology and its complications.

## Author Contributions

**Kathleen A. Stringer:** conceptualization, investigation, funding acquisition, writing – original draft, writing – review and editing, formal analysis, data curation, project administration. **David Goldberg:** investigation, writing – review and editing, data curation. **Sharon Chen:** investigation, writing – review and editing, data curation. **Philip Thrush:** investigation, writing – review and editing, data curation. **Eric M. Graham:** investigation, writing – review and editing, data curation. **Adam Lubert:** investigation, writing – review and editing, data curation. **Jeffrey Myers:** conceptualization, writing – review and editing. **Laura McLellan:** writing – review and editing, methodology, data curation. **Thomas Flott:** methodology, writing – review and editing, data curation. **Samya Nasr:** conceptualization, writing – original draft, methodology, writing – review and editing, data curation, funding acquisition, project administration. **Kurt R. Schumacher:** conceptualization, funding acquisition, writing – original draft, methodology, writing – review and editing, data curation, project administration.

## Conflicts of Interest

David Goldberg consults for Mezzion Pharmaceuticals and Inozyme Pharma. Kathleen Stringer is a scientific editor at *Pharmacotherapy*. All other authors declare no conflicts of interest.

## Supporting information


**Table S1:** Safety clinical laboratory values for each patient with plastic bronchitis.
**Table S2:** Median (IQR) spirometry values for patients with plastic bronchitis treated with inhaled tPA.
**Table S3:** Nonsafety adverse events during study drug treatment.
**Table S4:** Inclusion–exclusion criteria for control arm participants.
**Table S5:** Whole blood metabolites detected and named by ^1^H‐NMR spectroscopy.
**Table S6:** Serum immunoglobulin concentrations in PB patients.
**Figure S1:** Oxygen saturation (SpO_2_, %) as measured by pulse oximetry was lower in all surgically palliated congenital heart disease patients compared with healthy controls. The measurements for patients with plastic bronchitis are those acquired prior to inhaled tPA administration. The violin plots show the frequency distribution of the data and the individual data points. The large horizontal dashed lines represent the median and the small horizontal dashed lines represent the quartiles. ****p < 0.0001 (post‐ANOVA Holm‐Šídák's multiple comparisons test vs. healthy controls). Data represent 10/12 (83%) healthy controls, 8/9 (89%) Fontan controls, 8/8 (100%) plastic bronchitis patients, and 8/8 (100%) protein losing enteropathy (PLE) controls.
**Figure S2:** Comparison of spirometry values between healthy controls and participants with surgically palliated congenital heart disease (CHD) patients. Patients with plastic bronchitis (PB) were the most impaired as evidenced by a (A) lower forced vital capacity (FVC) and (B) forced expiratory volume in 1 s (FEV1) as well as a reduced FEV1/FVC ratio; all CHD participants had evidence of small airway dysfunction (D) as evidenced by a reduced FEF25%–75%. The violin plots show the frequency distribution of the data and the individual data points. The large horizontal dashed lines represent the median and the small horizontal dashed lines represent the quartiles. *p ≤ 0.05; **p ≤ 0.01; ***p ≤ 0.001(post‐ANOVA Holm‐Šídák's multiple comparisons test vs. healthy controls). Data represent 12/12 (100%) healthy controls, 9/9 (100%) Fontan controls, 8/8 (100%) plastic bronchitis patients, and 7/8 (88%) protein losing enteropathy (PLE) controls.
**Figure S3:** Inhaled tPA treatment did not improve Brasfield chest radiograph scores. Chest radiographs acquired in patients with plastic bronchitis (*n* = 8) prior to and following inhaled tPA treatment were scored using the Brasfield Criteria (see text in main manuscript). The green line at a score of 25 represents a normal chest radiograph.
**Figure S4:** Whole blood metabolomics differentiates patients with surgically palliated congenital heart disease (CHD) with protein losing enteropathy (PLE) and plastic bronchitis (PB) from healthy controls. (A) 2‐oxoisocaproate, also known as ketoleucine, a product branched chain amino acid metabolism, and (B) creatine, which facilitates the recycling of ATP, were altered in PLE patients compared with healthy controls. (C) Glutathione, the most abundant antioxidant in humans and (D) the metabolic by‐product, lactate, were altered in PB patients. (E) Pathway mapping of metabolites (A–D) identified four metabolic pathways that were disrupted in patients with complications of surgical palliated CHD. Violin plots show the frequency distribution of the data and the individual data points. The median is represented by the large horizontal dashed lines and the quartiles by the small horizontal dashed lines. *p ≤ 0.05; **p ≤ 0.01; (post‐ANOVA Holm‐Šídák's multiple comparisons test vs. healthy controls). PLE = protein losing enteropathy. Data represent 11/12 (92%) healthy controls, 8/9 (89%) Fontan controls, 6/8 (75%) plastic bronchitis patients, and 8/8 (100%) PLE controls.
**Figure S5:** Serum immunoglobulin (Ig) concentrations. There were no differences in (A) IgA or (B) IgM between patients with surgically palliated CHD and healthy controls. However, (C) IgG was lower in protein losing enteropathy (PLE) patients compared with healthy controls. Violin plots show the frequency distribution of the data and the individual data points. The median is represented by the large horizontal dashed lines and the quartiles by the small horizontal dashed lines. ****p ≤ 0.0001 (post‐ANOVA Holm‐Šídák's multiple comparisons test vs. healthy controls). Data represent 11/12 (92%) healthy controls, 9/9 (100%) Fontan controls, 8/8 (100%) plastic bronchitis patients, and 8/8 (100%) PLE controls.
**Figure S6:** Plasma tissue plasminogen activator (tPA) concentrations were (A) highly variable prior to and during inhaled tPA treatment. (B) There were no differences in plasma tPA concentrations between healthy controls and patients in the congenital heart disease groups (post‐ANOVA Holm‐Šídák's multiple comparisons test vs. healthy controls). Pretreatment tPA concentrations were used for patients with plastic bronchitis for this comparison. Data in panel (A) represent the median (IQR) *n* = 4 patients/time point except for day 3 (*n* = 2). Data in panel (B) represent 11/12 (92%) healthy controls, 8/9 (89%) Fontan controls, 4/8 (50%) plastic bronchitis patients, and 8/8 (100%) protein losing enteropathy (PLE) controls.

## Data Availability

All per protocol study measurements/end points are included in this manuscript and its supplement. Data will be shared in accordance with current NIH data sharing policies. De‐identified, individual participant data will be made available to investigators upon request to the corresponding author at stringek@umich.edu. Data request applications must provide an explanation for how the data will be used. The application will require review by and approval from the study's principal investigators (K.A.S. and K.R.S.) and a data use agreement.
