# Supplementary material for: A Multicenter, Open‐Label Study to Assess the Safety of Nebulized Tissue Plasminogen Activator for the Acute Treatment of Pediatric Plastic Bronchitis: The PLATyPuS Trial
Source: Pharmacotherapy. 2025 Sep 5;45(10):677–87. doi: 10.1002/phar.70056 (PMC12530010; doi:10.1002/phar.70056)
Supplement: Supplementary file 1 — Table S1: Safety clinical laboratory values for each patient with plastic bronchitis. Table S2: Median (IQR) spirometry values for patients with plastic bronchitis treated with inhaled tPA. Table S3: Nonsafety adverse events during study drug treatment. Table S4: Inclusion–exclusion criteria for control arm participants. Table S5: Whole blood metabolites detected and named by 1H‐NMR spectroscopy. Table S6: Serum immunoglobulin concentrations in PB patients. Figure S1: Oxygen saturation (SpO2, %) as measured by pulse oximetry was lower in all surgically palliated congenital heart disease patients compared with healthy controls. The measurements for patients with plastic bronchitis are those acquired prior to inhaled tPA administration. The violin plots show the frequency distribution of the data and the individual data points. The large horizontal dashed lines represent the median and the small horizontal dashed lines represent the quartiles. ****p < 0.0001 (post‐ANOVA Holm‐Šídák's multiple comparisons test vs. healthy controls). Data represent 10/12 (83%) healthy controls, 8/9 (89%) Fontan controls, 8/8 (100%) plastic bronchitis patients, and 8/8 (100%) protein losing enteropathy (PLE) controls. Figure S2: Comparison of spirometry values between healthy controls and participants with surgically palliated congenital heart disease (CHD) patients. Patients with plastic bronchitis (PB) were the most impaired as evidenced by a (A) lower forced vital capacity (FVC) and (B) forced expiratory volume in 1 s (FEV1) as well as a reduced FEV1/FVC ratio; all CHD participants had evidence of small airway dysfunction (D) as evidenced by a reduced FEF25%–75%. The violin plots show the frequency distribution of the data and the individual data points. The large horizontal dashed lines represent the median and the small horizontal dashed lines represent the quartiles. *p ≤ 0.05; **p ≤ 0.01; ***p ≤ 0.001(post‐ANOVA Holm‐Šídák's multiple comparisons test vs. healthy controls). Data rep [file PHAR-45-677-s001.docx]

**SUPPLEMENTARY INFORMATION**

A Multi-center, Open-label Study to Assess the Safety of Nebulized Tissue Plasminogen Activator for the Acute Treatment of Pediatric Plastic Bronchitis: the PLATyPuS Trial

This was an open-label, multicenter clinical trial of inhaled tPA for the treatment of acute exacerbations of plastic bronchitis (PB). The objectives of the trial were to: (i) test the safety and efficacy of an inhaled tPA regimen in children with PB; and (ii) identify potential candidate biomarkers of inhaled tPA drug response. Safety end points consisted of the development of new, active bleeding that is systemic and/or pulmonary and/or new hematuria (defined as gross hematuria). Secondary end points of efficacy were also measured (e.g., frequency of cast production). Urine and blood were also collected for the development of potential biomarkers of inhaled tPA drug response.

**Methods**

*Human Subjects*. This was an open-label study of up to 13 patients with PB that served as their own controls. A planned enrollment of a group of healthy subjects (n=12), Fontan patients without PB (n=12), and Fontan patients with protein losing enteropathy (PLE) (n=12) served as controls for biomarker assay development. The active treatment arm (inhaled tPA) was conducted across six clinical centers (these centers also enrolled up to two PLE control patients). All other control subjects were only enrolled at the University of Michigan. The inclusion-exclusion criteria for control arm participants are shown in **Supplementary Table 4**.

*Sample Size:* The original planned enrollment for tPA-treated patients was 24. However, based on a safety assessment following the enrollment and treatment of six PB patients, no tPA-treated patient reached a study safety end point (e.g., systemic bleeding). Given this and ongoing difficulty with study enrollment, the planned study enrollment was adjusted to 11 PB patients. Specifically, considering the 0% observed rate of systemic bleeding in the six PB patients who have completed the study, we assumed the same bleeding incidence rate for subsequent enrolled PB patient. As such, enrollment of a total of 11 to 13 patients with PB with no participant experiencing systemic bleeding, with 95% confidence, the population bleeding incidence was expected to be within 0% - 28.5% to 0% - 24.7% intervals. Therefore, the previously tolerated upper 95% confidence limit of 25% with a sample size of 13 would be maintained and modestly increase this limit with enrollment of 11 PB patient.

*Study Drug Administration.* In advance of study drug administration, alteplase (50-mg vial) was reconstituted with sterile water for injection (USP; 50 ml) and 5 ml was withdrawn from the vial using a 10-mL syringe. At the time of dosing, study drug was transferred into and administered using a standard nebulizer (NebuTech HDN nebulizer, Ref 8960-7; Salter Labs, SunMed Medical Solutions, Marlton, NJ, USA) and the hospital’s internal compressed oxygen system via a flow meter (8 LPM) by a respiratory therapist. Administration of study drug took approximately 10 minutes. Continued “off protocol” administration of inhaled tPA following the trial’s 72-hour drug administration was permitted at the discretion of the treating physician. Oscillatory chest physiotherapy vests and/or bronchodilators were not permitted within 2 hours before or after inhaled tPA administration. Co-administration of other medications with tPA were not permitted.

*Additional secondary outcomes*. To gather general efficacy information about inhaled tPA treatment efficacy, the frequency of production/expectoration and size of airway casts (weight and length) following tPA treatment was assessed in PB patients; this was self-reported. The requirement for urgent or emergent bronchoscopy and/or mechanical ventilation was also measured as a metric of progression of the PB exacerbation. Fibrin and mucin content was determined as part of the histopathological assessment of expectorated casts as previously described.^1^ Measurements of patient-centered outcome were made using the Cystic Fibrosis Questionnaire Revised (CFQ-R; https://qol.thoracic.org/sections/instruments/ae/pages/cfq-cfq-r.html)^2^ prior to tPA treatment, at hospital discharge and at the 30-day follow up visit.

*Tertiary outcome*. Whole blood (sodium heparin preserved) and urine (clean catch if feasible) were collected from PB patients prior to tPA treatment, at least once during treatment and then again at hospital discharge. A single blood and urine sample were obtained from the control participants. These samples were for research purposes only and were collected following a standard operating procedure (SOP) that was developed in advance of the initiation of the trial.

Metabolomics data were generated using the established protocols in the University of Michigan NMR Metabolomics Laboratory as previously described.^3^

Spectra were acquired at the University of Michigan’s Biochemical NMR Core Laboratory on a Varian (now Agilent, Inc., Santa Clara, CA) 500MHz NMR spectrometer with a VNMRS console operated by host software VNMRJ 4.0. Spectra were acquired at room temperature (295.45+/-0.3K) using a 5-mm Agilent "One-probe" as previously described.^3^ NMR spectra were analyzed using Chenomx NMR Suite V10.0 (Chenomx, Inc., Edmonton, AB, Canada).

All spectra were analyzed by a single researcher blinded to experimental group and time point. Using Metaboanalyst, missing values were imputed as 1/5^th^ the lowest value of the representative metabolite concentration.^4^ Data were then log-transformed and mean centered to achieve a normal distribution for parametric statistics. The metabolic pathway relatedness of found differentiating metabolites was determined using Metscape^5^, a plugin application for Cytoscape.^6^

*Other measurements.* Patients with Fontan physiology are prone to loss of immunoglobulin (Ig).^7^ Three Ig (A, M, and G) were measured by each site’s respective clinical laboratory prior to tPA treatment, at hospital discharge and at the 30-day follow up visit. Blood samples were also collected for the measurement of tPA concentration prior to, during, and after treatment (hospital discharge). Plasma tPA total antigen concentrations were measured by ELISA (Innovative Research, kit IHUTPAKTT, Novi, MI USA) in accordance with the manufacturer’s instructions. Blood samples for Ig and tPA measurements were also collected as part of each control participant’s single research visit.

**Results**

*Primary outcomes.* One patient had a pre-treatment level of fibrin degradation products (FDP) reported as > 5 µg/mL but this was determined not to be clinically significant since all subsequent values were ≤ 5 µg/mL (**Supplementary Table 1**). Furthermore, elevations in FDP were not viewed as study drug related since there was no evidence of fibrinogen depletion. All fibrinogen blood concentrations during inhaled tPA treatment remained in the reference range. This is consistent with blood levels to total antigen tPA which did not change during the course of treatment (see other measurements section below).

*Additional secondary outcomes.* None of the enrolled PB patients expectorated airway casts during tPA treatment so the frequency of production/expectoration, size of airway casts (weight and length), and fibrin and mucin content could not be assessed. None of the PB patients required urgent or emergent bronchoscopy and/or mechanical ventilation. Due to insufficient reporting of cystic fibrosis questionnaire-revised (CFQ-R) data, an assessment of patient-centered outcomes could not be made.

*Tertiary outcome.* Given the study’s small sample size, the complexity of the urine metabolome^8^, and that too few (≤ 2) samples were collected at most time points for meaningful interpretation, only baseline whole blood specimens were analyzed. A total of 38 metabolites were identified and quantified (**Supplementary Table 5**). Of these, two and five metabolites differentiated PB and PLE patients from healthy controls, respectively (**Supplementary Figure 4A-G**). These metabolites are associated with several different metabolic pathways as identified by Metscape (**Supplementary Figure 4H**).

*Other measurements*. Immunoglobulin (Ig) A, M, and G concentrations did not change from baseline values (**Supplementary Table 6**) during or after tPA treatment. Pre-treatment values were also not different from healthy controls (**Supplementary** **Figure 5**) but IgG was lower in PLE controls compared with PB patients. Plasma concentrations of tPA were highly variable and did not change during the course of inhaled tPA administration. However, plasma samples were only available for approximately 50% of the PB patients (**Supplementary Figure 6A**). There were no differences in plasma tPA concentration across the participant groups (**Supplementary Figure 6B**).

| *HEMATOCRIT (%)* | Pre-treatment | Treatment Day 1 | Treatment Day 2 | Treatment Day 3 | Hospital Discharge | 30d close out visit |
| --- | --- | --- | --- | --- | --- | --- |
| PB1 | 42 |  | 43.3 | 43.7 | 43.8 | 43.1 |
| PB2 | 43.1 | 41 | 37.8 |  |  |  |
| PB3 | 47.5 | 48.6 | 46.3 | 44.4 | 38.3 | 43.2 |
| PB4 | 41.8 | 45.9 | 44.3 | 42.9 | 37.6 |  |
| PB5 | 42.2 | 46.5 | 42.1 | 42 | 42.9 |  |
| PB6 | 40.9 |  | 47.9 | 48.9 | 44.9 | 32.4 |
| PB7 | 46.7 |  | 49.3 | 48.3 | 49.4 | 44.7 |
| PB8 | 48.3 | 46.6 | 44.9 | 44.5 | 43.9 | 31.1 |
| *PLATELETS (x 10^5^ cells/µL)* | |  |  |  |  |  |
| PB1 | 193 |  | 201 | 212 | 214 | 183 |
| PB2 | 209 | 197 | 164 |  |  |  |
| PB3 | 339 | 381 | 350 | 333 | 181 | 144 |
| PB4 | 337 | 374 | 353 | 331 | 205 |  |
| PB5 | 206 | 202 | 188 | 203 | 191 |  |
| PB6 | 109 |  | 174 | 160 | 168 | 81 |
| PB7 | 180 |  | 246 | 273 | 369 | 319 |
| PB8 | 179 | 173 | 166 | 145 | 157 | 150 |
| *FIBRINOGEN (mg/dL)* | |  |  |  |  |  |
| PB1 | 294 |  | 285 | 291 | 325 | 404 |
| PB2 | 246 | 254 | 251 |  |  |  |
| PB3 | 389 | 413 | 341 | 384 | 567 | 345 |
| PB4 | 321 | 345 | 337 | 292 | 413 |  |
| PB5 | 269 | 290 | 229 | 239 | 309 |  |
| PB6 | 359 |  | 343 | 391 | 337 | 288 |
| PB7 |  |  | 408 | 440 | 520 | 441 |
| PB8 | 312 | 329 | 354 | 254 | 358 | 124 |
| *FIBRIN DEGRADATION PRODUCTS (µg/mL)* | | |  |  |  |  |
| PB1 | 0.27 |  | 0.26 | 0.27 | 0.27 | 0.61 |
| PB2 | 0.29 | 0.27 | 0.27 |  |  |  |
| PB3 | 0.46 | 0.61 | 0.4 | 3.94 | 10.37 | 0.53 |
| PB4 | 0.27 | 0.27 | 0.31 | 0.5 | 6 |  |
| PB5 | 0.326 | 0.306 | 0.296 | 0.312 | 4.526 |  |
| *PB6 | *> 5* |  | *< 5* | *< 5* | *< 5* | *< 5* |
| *PB7 |  |  | *5** | *5** | *5** | *5** |
| PB8 | 0.32 | 0.22 | 0.24 | 0.26 | 0.53 | 3.63 |

**Supplementary Table 1**: Safety clinical laboratory values for each patient with plastic bronchitis

| *APTT (s)* | Pre-treatment | Treatment Day 1 | Treatment Day 2 | Treatment Day 3 | Hospital Discharge | 30d close out visit |
| --- | --- | --- | --- | --- | --- | --- |
| PB1 | 33.6 |  | 33.3 | 32.9 | 31.2 | 29.2 |
| PB2 | 29.2 | 32.6 | 31.4 |  |  |  |
| PB3 | 38.7 | 37.9 | 38.5 | 33.5 | 32.1 | 36.9 |
| PB4 | 41.8 | 38.8 | 41.8 | 41.5 | 34.8 |  |
| PB5 | 31.8 | 33.9 | 32.9 | 31.3 | 25.7 |  |
| PB6 | 27.4 |  | 27.3 | 27.7 | 26.7 | 29.5 |
| PB7 |  |  | 31.7 | 31.2 | 34.6 | 36.5 |
| PB8 | 23.7 | 26.1 | 26.1 | 33.7 | 26.7 | 21.2 |
| *INR* |  |  |  |  |  |  |
| PB1 | 1.62 |  | 1.47 | 1.27 | 1.2 | 1.18 |
| PB2 | 0.97 | 1.1 | 1.04 |  |  |  |
| PB3 | 1.2 | 1.19 | 1.26 | 1.39 | 1.14 | 1.21 |
| PB4 | 1.34 | 1.33 | 1.31 | 1.25 | 1.26 |  |
| PB5 | 1.27 | 1.22 | 1.28 | 1.26 | 1.7 |  |
| PB6 | 1.1 |  | 1.1 | 1.1 | 1.2 | 1.1 |
| PB7 |  |  | 1.2 | 1 | 1 | 1.1 |
| PB8 | 1.1 | 1.1 | 1.1 | 1.2 | 1.2 | 1.1 |

*these clinical assays were qualitative and were not included in the mean (SD) calculation for Figure 2.

APTT=activated partial thromboplastin time; INR=international normalized ratio

**Supplementary Table 2**: Median (IQR) spirometry values for patients with plastic bronchitis treated with inhaled tPA

| Timepoint | Study Days | **Median FEV1 (% predicted)** | Upper Limit | Lower Limit | Patients (n) | Measurements (n) |
| --- | --- | --- | --- | --- | --- | --- |
| Baseline | 0 | 65 | 99 | 28 | 3 | 3 |
| Treatment (all timepoints) | 3 | 50 | 68 | 44 | 7 | 13 |
| Post-Treatment (Hx d/c + 30d) | 10 | 48 | 51 | 38 | 6 | 7 |
|  |  |  |  |  |  |  |
| Timepoint | Days | **Median FVC (% predicted)** | Upper Limit | Lower Limit | Patients (n) | Measurements (n) |
| Baseline | 0 | 76 | 96 | 24 | 3 | 3 |
| Treatment (all timepoints) | 3 | 55 | 73 | 39 | 7 | 13 |
| Post-Treatment (Hx d/c + 30d) | 10 | 52 | 70 | 36 | 6 | 7 |
|  |  |  |  |  |  |  |
| Timepoint | Days | **Median FEV1/FVC ratio (%predicted)** | Upper Limit | Lower Limit | Patients (n) | Measurements (n) |
| Baseline | 0 | 103 | 114 | 85 | 3 | 3 |
| Treatment (all timepoints) | 3 | 94 | 105 | 86 | 7 | 13 |
| Post-Treatment (Hx d/c + 30d) | 10 | 91 | 101 | 82 | 6 | 7 |
|  |  |  |  |  |  |  |
| Timepoint | Days | **Median FEF25-75%**  **(% predicted)** | Upper Limit | Lower Limit | Patients (n) | Measurements (n) |
| Baseline | 0 | 41 | 46 | 36 | 2 | 2 |
| Treatment (all timepoints) | 3 | 46 | 67 | 39 | 7 | 13 |
| Post-Treatment (Hx d/c + 30d) | 10 | 35 | 41 | 28 | 6 | 7 |

The median (IQR) spirometry values were calculated from the number of measurements at each time point; Hx d/c = hospital discharge; FEV1=forced expiratory volume in 1 second; FVC=forced vital capacity: FEF25-75%=forced mid-expiratory flow. These data are presented as plots in Figure 3 of the main manuscript.

**Supplementary Table 3:** Non-Safety Adverse Events During Study Drug Treatment

| *Study drug related* | | |
| --- | --- | --- |
| **System Organ Classification** | **AE Description (Verbatim Term)** | **Outcome** |
| Respiratory, thoracic, and mediastinal disorders | Coughing | Recovered / Resolved |
| Respiratory, thoracic, and mediastinal disorders | Coughing | Recovered / Resolved |
| Respiratory, thoracic, and mediastinal disorders | Coughing | Recovered / Resolved |
| Nervous system disorder | Dizziness | Recovered/ Resolved |
| Respiratory, thoracic, and mediastinal disorders | Hypoxemia | Recovered/ Resolved |
| Respiratory, thoracic, and mediastinal disorders | Hypoxemia | Recovered/ Resolved |
| Gastrointestinal disorders | Emesis | Drug Withdrawn* |
| *Not study drug related* | | |
| **System Organ Classification** | **AE Description (Verbatim Term)** | **Outcome** |
| Cardiac disorders | Atrial tachycardia | Recovered / Resolved |
| General disorder | Fever | Recovered / Resolved |
| Respiratory, thoracic, and mediastinal disorders | Rhinovirus | Recovered / Resolved |
| Respiratory, thoracic, and mediastinal disorders | Coughing | Recovered / Resolved |
| Vascular disorders | Low Blood Pressure | Recovered / Resolved |
| Respiratory, thoracic, and mediastinal disorders | Patchy Lobe Atelectasis | Recovered / Resolved |
| Coagulation | Elevated D-Dimer | Recovered / Resolved |
| Immunological | Elevated WBC Count | Recovered / Resolved |
| Gastrointestinal disorders | Abdominal Pain | Recovered / Resolved |
| Gastrointestinal disorders | Poor appetite and constipation | Recovered / Resolved |

*this patient also experienced epistaxis which prompted study drug discontinuation;

AE=adverse event; WBC=white blood cell

**Supplementary Table 4**: Inclusion-exclusion criteria for control arm participants

### ***Inclusion Criteria***

### Healthy Controls

- Healthy children ≥ 5 years of age but ≤18 years of age with no other underlying concomitant illness or chronic medication use (with the exception of vitamin supplements)

### non-PB Fontan Controls

- Children ≥ 5 years of age but ≤18 years of age with uncomplicated Fontan physiology with no history of PB, other Fontan-associated complications (e.g., hepatopathy, PLE), or other concomitant illnesses (e.g., asthma).

### PLE Fontan Controls

- Children ≥ 5 years of age but ≤18 years of age with Fontan physiology, no history of PB, and a diagnosis of PLE defined as clinically symptomatic hypoproteinemia and/or enteral protein loss.

All participants had to weigh at least 18.6 kg (41 lbs)

### ***Exclusion Criteria for Healthy, non-PB Fontan Controls and PLE Fontan Controls***

1. Exceeds the 100th percentile for body weight or has a BMI greater than 30 kg/m^2^.
2. History of post-operative chylothorax following any palliation surgery (except for PLE controls).
3. Liver dysfunction (ALT & AST) (defined as ≥ 3X the normal levels of one or both liver transaminases, ALT & AST)
4. COVID-19 positive within the last 14 days prior to the scheduled visit and/or the presence of symptoms consistent with COVID-19 at the time of the visit
5. Suspected or active concurrent infectious illness

PB=plastic bronchitis; PLE=protein losing enteropathy; ALT=alanine aminotransferase; AST=aspartate aminotransferase; COVID-19=novel coronavirus disease-2019

|  | KEGG ID | HMDB ID | Compound Name |
| --- | --- | --- | --- |
| 1 | C05984 | HMDB00008 | **2-Hydroxybutyrate** |
| 2 | C00233 | HMDB00695 | **2-Oxoisocaproate** |
| 3 | C01089 | HMDB00357 | **3-Hydroxybutyrate** |
| 4 | C00008 | HMDB01341 | **ADP** |
| 5 | C00020 | HMDB00045 | **AMP** |
| 6 | C00002 | HMDB00538 | **ATP** |
| 7 | C00041 | HMDB00161 | **Alanine** |
| 8 | C00152 | HMDB00168 | **Asparagine** |
| 9 | C00049 | HMDB00191 | **Aspartate** |
| 10 | C00719 | HMDB00043 | **Betaine** |
| 11 | C00318 | HMDB00062 | **Carnitine** |
| 12 | C00114 | HMDB00097 | **Choline** |
| 13 | C00158 | HMDB00094 | **Citrate** |
| 14 | C00300 | HMDB00064 | **Creatine** |
| 15 | C00791 | HMDB00562 | **Creatinine** |
| 16 | C00031 | HMDB00122 | **Glucose** |
| 17 | C00025 | HMDB00148 | **Glutamate** |
| 18 | C00064 | HMDB00641 | **Glutamine** |
| 19 | C00051 | HMDB00125 | **Glutathione** |
| 20 | C00116 | HMDB00131 | **Glycerol** |
| 21 | C00037 | HMDB00123 | **Glycine** |
| 22 | C00135 | HMDB00177 | **Histidine** |
| 23 | C00407 | HMDB00172 | **Isoleucine** |
| 24 | C00186 | HMDB00190 | **Lactate** |
| 25 | C00123 | HMDB00687 | **Leucine** |
| 26 | C00047 | HMDB00182 | **Lysine** |
| 27 | C00073 | HMDB00696 | **Methionine** |
| 28 | C02571 | HMDB00201 | **O-Acetylcarnitine** |
| 29 | C00077 | HMDB00214 | **Ornithine** |
| 30 | C00079 | HMDB00159 | **Phenylalanine** |
| 31 | C00148 | HMDB00162 | **Proline** |
| 32 | C01879 | HMDB00267 | **Pyroglutamate** |
| 33 | C00065 | HMDB00187 | **Serine** |
| 34 | C00245 | HMDB00251 | **Taurine** |
| 35 | C00188 | HMDB00167 | **Threonine** |
| 36 | C01104 | HMDB00925 | **Trimethylamine N-oxide** |
| 37 | C00082 | HMDB00158 | **Tyrosine** |
| 38 | C00183 | HMDB00883 | **Valine** |

**Supplementary Table 5**: Whole Blood Metabolites Detected and Named by ^1^H-NMR Spectroscopy

^1^H-NMR=proton nuclear magnetic resonance; KEGG=Kyoto Encyclopedia of Genes and Genomes (<https://www.genome.jp/kegg/>); HMDB= Human Metabolome Database (<https://hmdb.ca/>)

**Supplementary Table 6**: Serum immunoglobulin concentrations in PB patients.

|  | IgA | | | IgM | | | IgG | | |
| --- | --- | --- | --- | --- | --- | --- | --- | --- | --- |
| Time point | mean | SD | n | mean | SD | n | mean | SD | n |
| Pre-treatment | 127 | 50 | 8 | 114 | 71 | 8 | 834 | 400 | 8 |
| Post-treatment | 128 | 45 | 6 | 125 | 92 | 6 | 874 | 420 | 6 |
| Follow-up | 123 | 54 | 5 | 149 | 53 | 5 | 896 | 564 | 5 |

Ig=immunoglobulin; PB=plastic bronchitis; SD=standard deviation


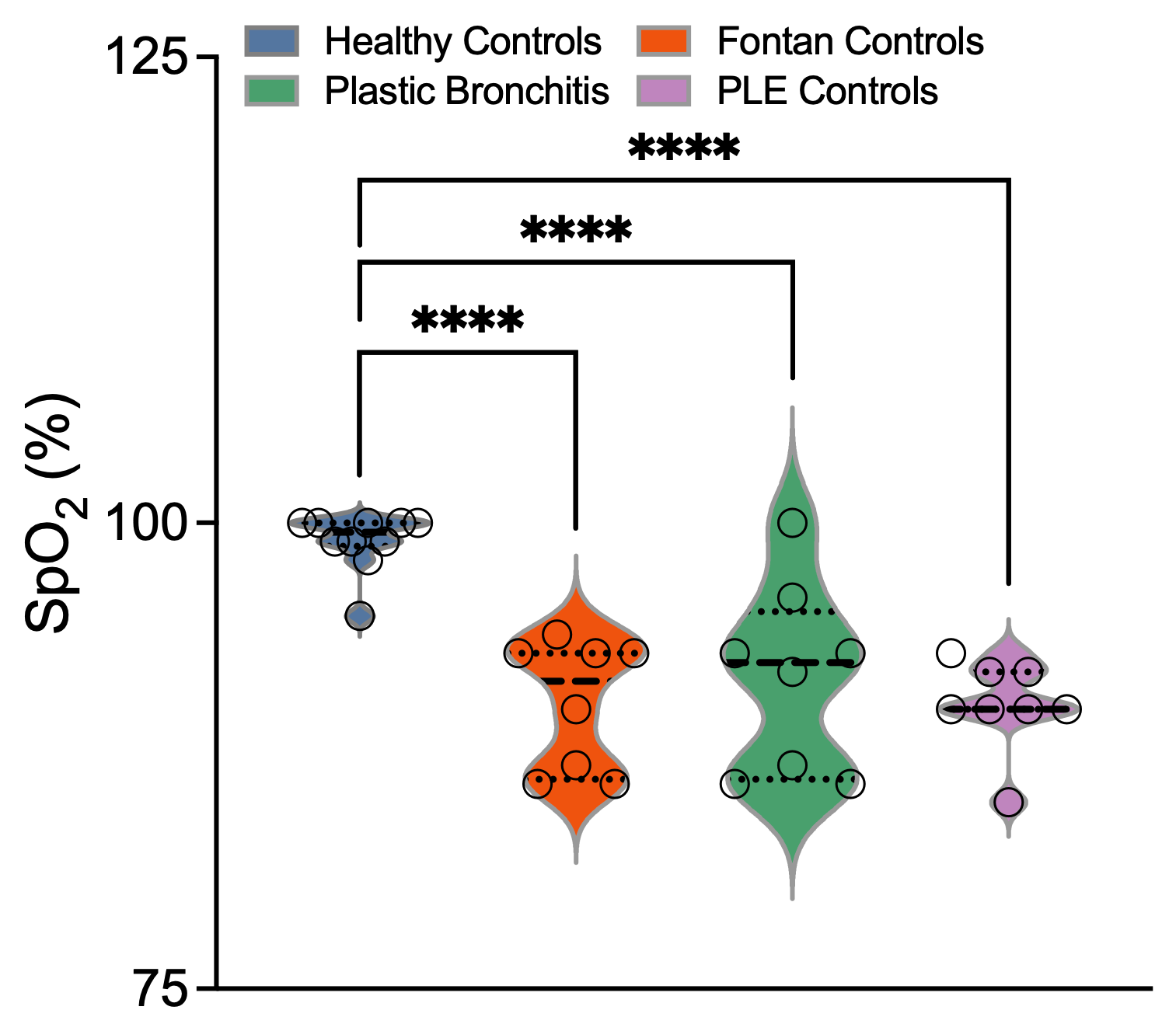


**Supplementary Figure 1**. Oxygen saturation (SpO_2,_ %) as measured by pulse oximetry was lower in all surgically palliated congenital heart disease patients compared with healthy controls. The measurements for patients with plastic bronchitis are those acquired prior to inhaled tPA administration. The violin plots show the frequency distribution of the data and the individual data points. The large horizontal dashed lines represent the median and the small horizontal dashed lines represent the quartiles. ****p<0.0001 (post-ANOVA Holm-Šídák's multiple comparisons test vs. healthy controls). Data represent 10/12 (83%) healthy controls, 8/9 (89%) Fontan controls, 8/8 (100%) plastic bronchitis patients, and 8/8 (100%) protein losing enteropathy (PLE) controls.

**
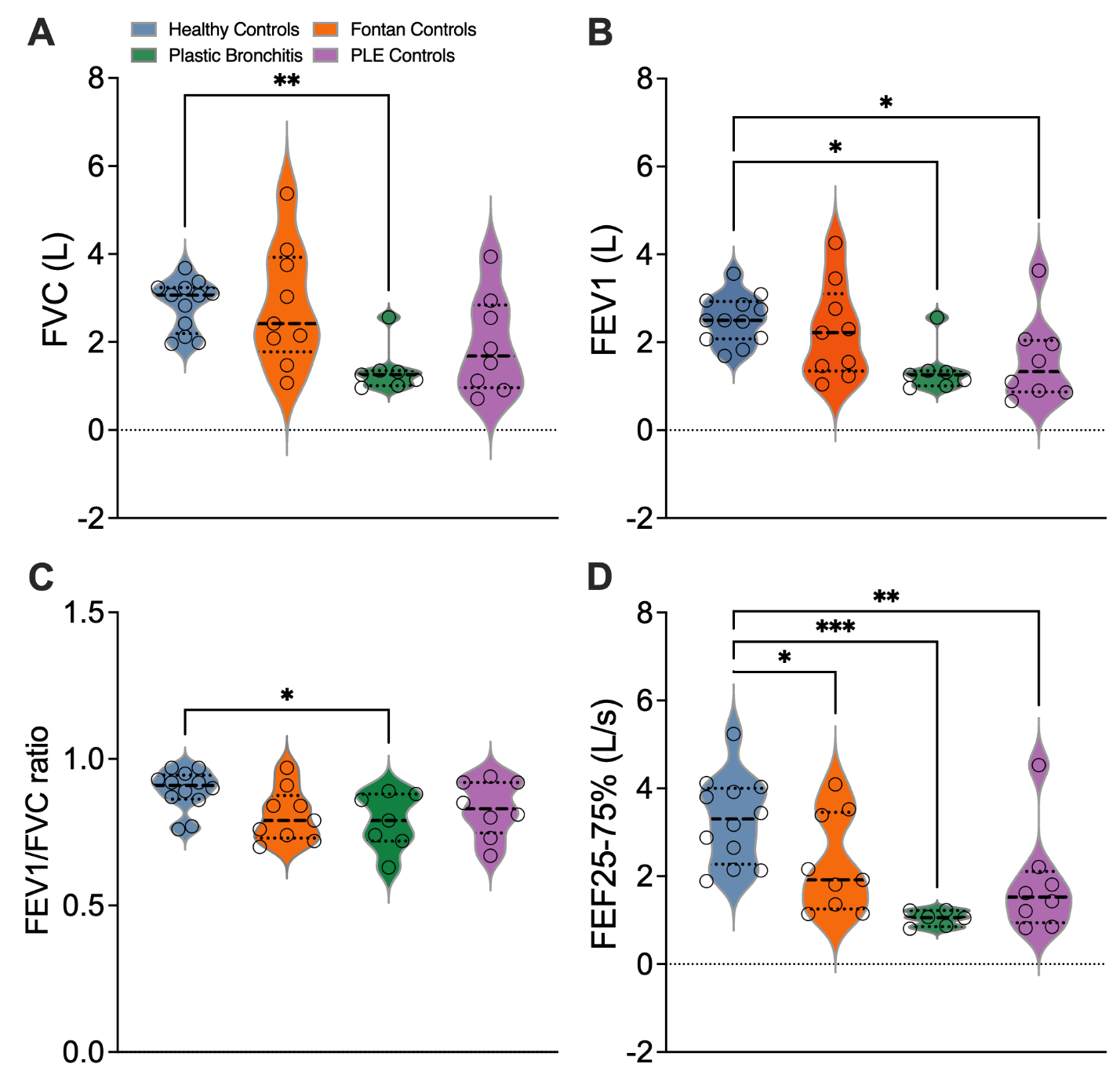
**

**Supplementary Figure 2**. Comparison of spirometry values between healthy controls and participants with surgically palliated congenital heart disease (CHD) patients. Patients with plastic bronchitis (PB) were the most impaired as evidenced by a (**A**) lower forced vital capacity (FVC) and (**B**) forced expiratory volume in 1 second (FEV1) as well as a reduced FEV1/FVC ratio; all CHD participants had evidence of small airway dysfunction (**D**) as evidenced by a reduced FEF25-75%. The violin plots show the frequency distribution of the data and the individual data points. The large horizontal dashed lines represent the median and the small horizontal dashed lines represent the quartiles. *p≤0.05; **p≤0.01; ***p≤0.001(post-ANOVA Holm-Šídák's multiple comparisons test vs. healthy controls). Data represent 12/12 (100%) healthy controls, 9/9 (100%) Fontan controls, 8/8 (100%) plastic bronchitis patients, and 7/8 (88%) protein losing enteropathy (PLE) controls.

**
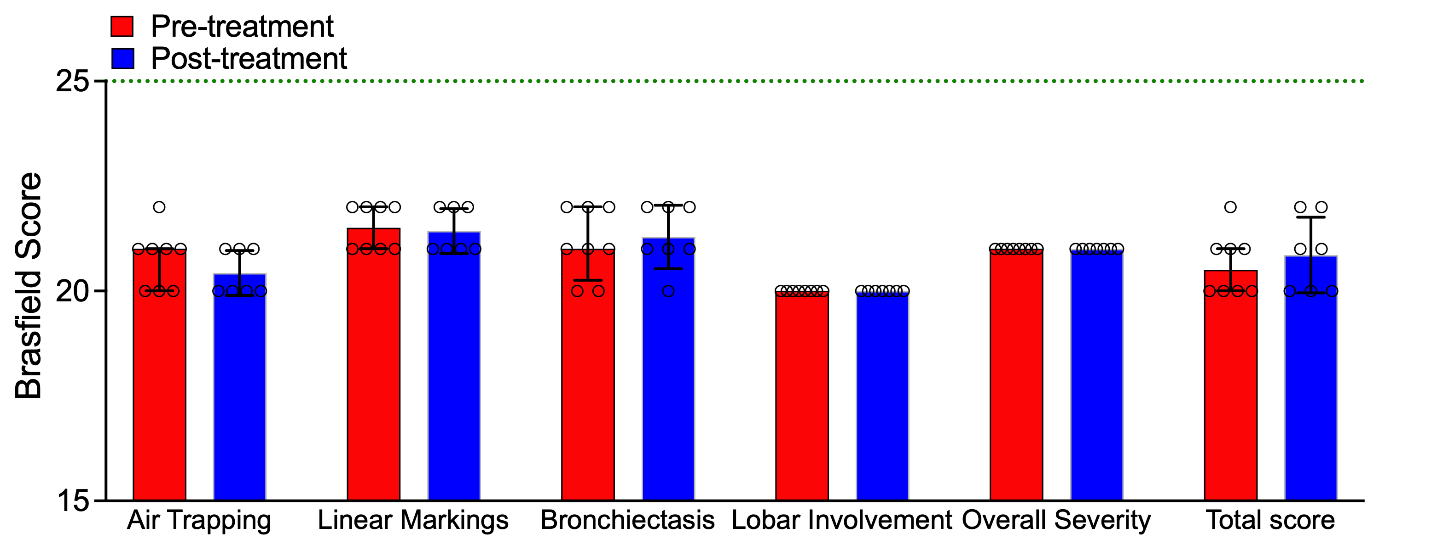
**

**Supplementary Figure 3**. Inhaled tPA treatment did not improve Brasfield chest radiograph scores. Chest radiographs acquired in patients with plastic bronchitis (n=8) prior to and following inhaled tPA treatment were scored using the Brasfield Criteria (see text in main manuscript). The green line at a score of 25 represents a normal chest radiograph.

**
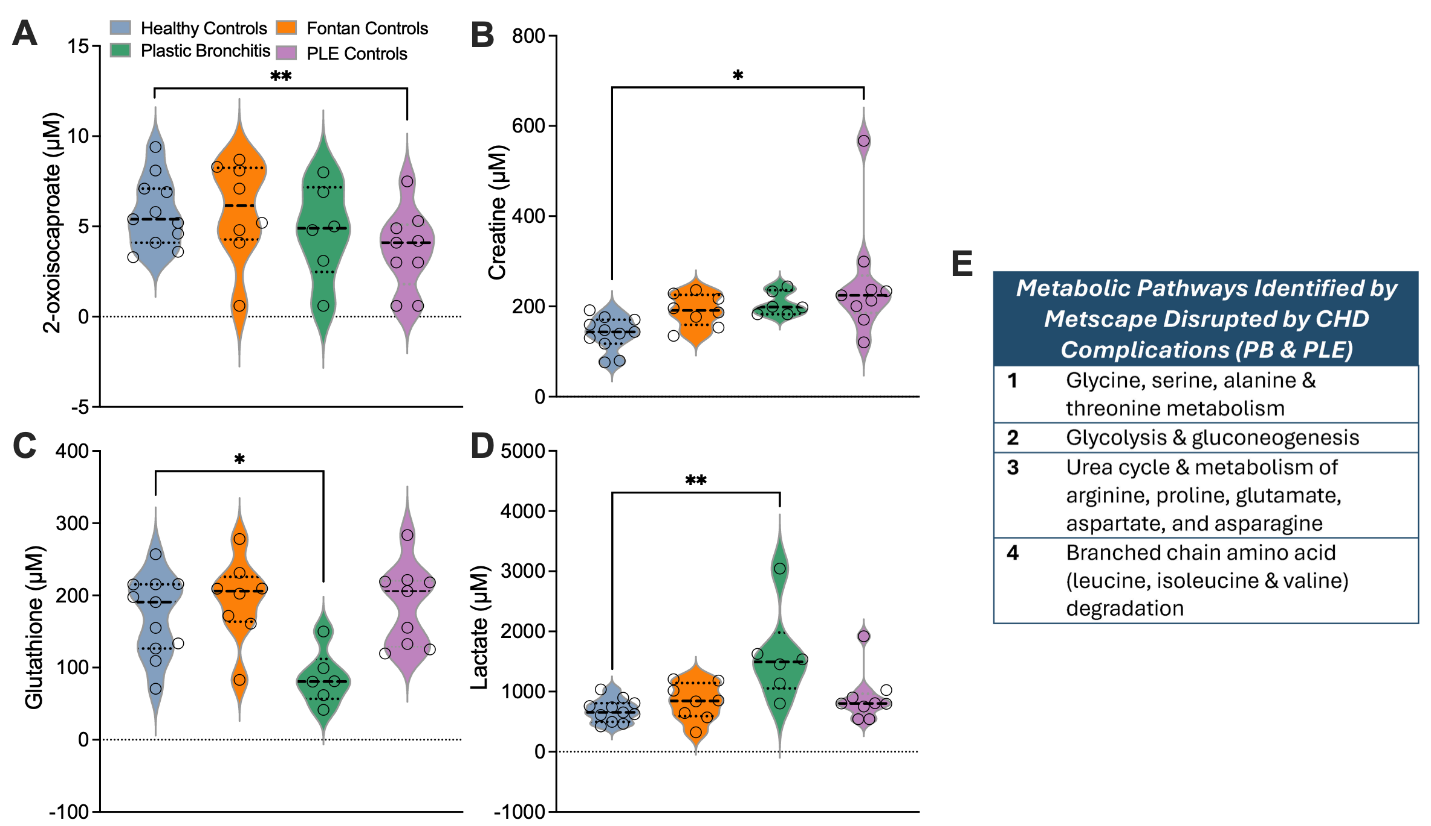
**

**Supplementary Figure 4**. Whole blood metabolomics differentiates patients with surgically palliated congenital heart disease (CHD) with protein losing enteropathy (PLE) and plastic bronchitis (PB) from healthy controls. (**A**) 2-oxoisocaproate, also known as ketoleucine, a product branched chain amino acid metabolism, and (**B**) creatine, which facilitates the recycling of ATP, were altered in PLE patients compared with healthy controls. (**C**) Glutathione, the most abundant antioxidant in humans and (**D**) the metabolic by-product, lactate, were altered in PB patients. (**E**) Pathway mapping of metabolites (**A-D**) identified four metabolic pathways that were disrupted in patients with complications of surgical palliated CHD. Violin plots show the frequency distribution of the data and the individual data points. The median is represented by the large horizontal dashed lines and the quartiles by the small horizontal dashed lines. *p≤0.05; **p≤0.01; (post-ANOVA Holm-Šídák's multiple comparisons test vs. healthy controls). PLE = protein losing enteropathy. Data represent 11/12 (92%) healthy controls, 8/9 (89%) Fontan controls, 6/8 (75%) plastic bronchitis patients, and 8/8 (100%) PLE controls.

**
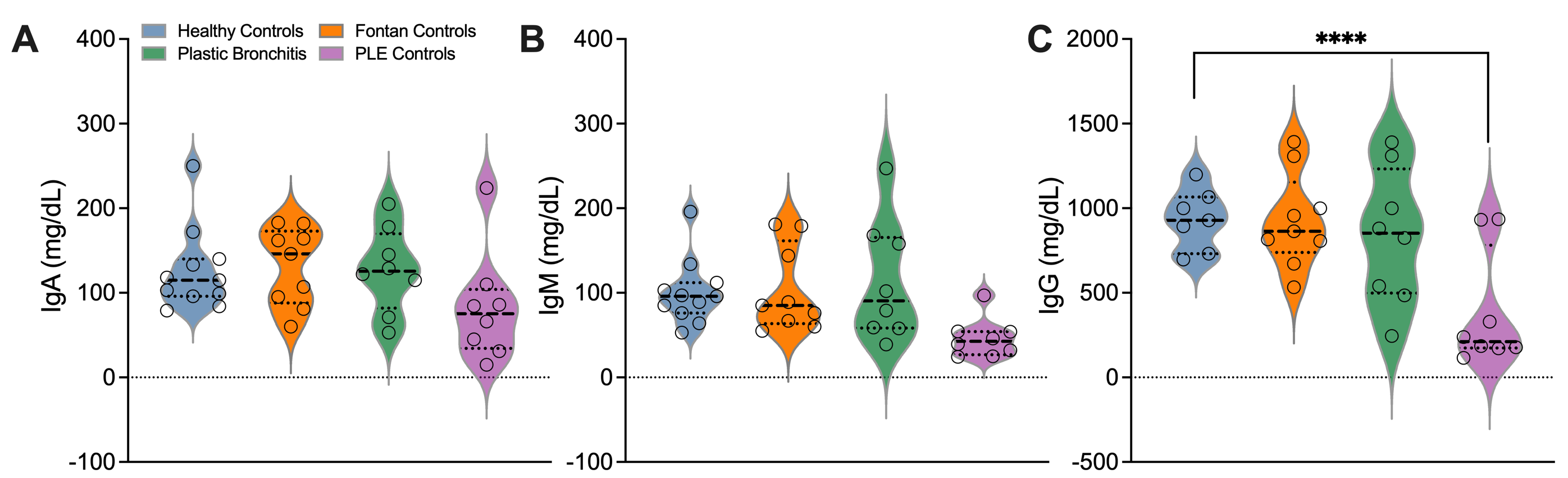
**

**Supplementary Figure 5**. Serum immunoglobulin (Ig) concentrations. There were no differences in (**A**) IgA or (**B**) IgM between patients with surgically palliated CHD and healthy controls. However, (**C**) IgG was lower in protein losing enteropathy (PLE) patients compared with healthy controls. Violin plots show the frequency distribution of the data and the individual data points. The median is represented by the large horizontal dashed lines and the quartiles by the small horizontal dashed lines. ****p≤0.0001 (post-ANOVA Holm-Šídák's multiple comparisons test vs. healthy controls). Data represent 11/12 (92%) healthy controls, 9/9 (100%) Fontan controls, 8/8 (100%) plastic bronchitis patients, and 8/8 (100%) PLE controls.

**
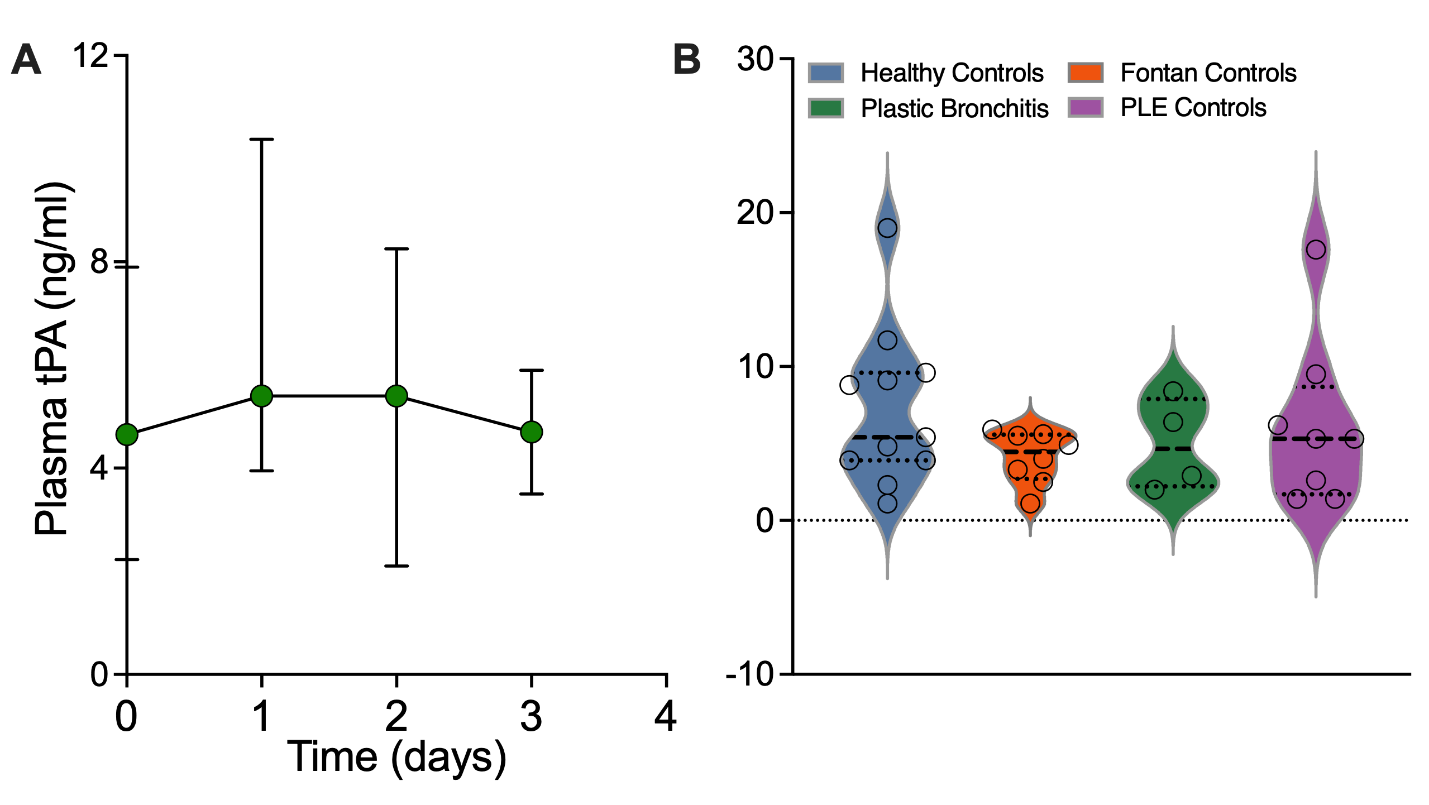
**

**Supplementary Figure 6**. Plasma tissue plasminogen activator (tPA) concentrations were (**A**) highly variable prior to and during inhaled tPA treatment. (**B**) There were no differences in plasma tPA concentrations between healthy controls and patients in the congenital heart disease groups (post-ANOVA Holm-Šídák's multiple comparisons test vs. healthy controls). Pre-treatment tPA concentrations were used for patients with plastic bronchitis for this comparison. Data in panel (**A**) represent the median (IQR) n=4 patients/time point except for day 3 (n=2). Data in panel (**B**) represent 11/12 (92%) healthy controls, 8/9 (89%) Fontan controls, 4/8 (50%) plastic bronchitis patients, and 8/8 (100%) protein losing enteropathy (PLE) controls.

**References**

1. Heath L, Ling S, Racz J, et al. Prospective, longitudinal study of plastic bronchitis cast pathology and responsiveness to tissue plasminogen activator. Pediatr Cardiol 2011;8:1182-9.

2. Solomon GM, Linnemann RW, Rich R, et al. Evaluation of elexacaftor-tezacaftor-ivacaftor treatment in individuals with cystic fibrosis and CFTR(N1303K) in the USA: a prospective, multicentre, open-label, single-arm trial. Lancet Respir Med 2024;12:947-57.

3. McHugh CE, Flott TL, Schooff CR, et al. Rapid, Reproducible, Quantifiable NMR Metabolomics: Methanol and Methanol: Chloroform Precipitation for Removal of Macromolecules in Serum and Whole Blood. Metabolites 2018;4.

4. Rubin BK. Plastic bronchitis: Casting a wider net. Pediatr Pulmonol 2023;11:3001-02.

5. Karnovsky A, Weymouth T, Hull T, et al. Metscape 2 bioinformatics tool for the analysis and visualization of metabolomics and gene expression data. Bioinformatics 2012;3:373-80.

6. Otasek D, Morris JH, Boucas J, Pico AR, Demchak B. Cytoscape Automation: empowering workflow-based network analysis. Genome Biol 2019;1:185.

7. Leroy M, Weis A, Backhoff D, Santibanez S, Mankertz A, Jux C. Isolated loss of vaccine immunity in the protein losings syndrome in a patient with a reverse one and a half ventricle palliation ("failing Fontan-like physiology"). Cardiol Young 2025;1-3.

8. Bouatra S, Aziat F, Mandal R, et al. The human urine metabolome. PLoS One 2013;9:e73076.
